# LAB-to-FAB Transition of 2D FETs: Available Strategies and Future Trends

**DOI:** 10.3390/nano14151237

**Published:** 2024-07-23

**Authors:** Yury Illarionov, Yezhu Lv, Yehao Wu, Yajing Chai

**Affiliations:** Laboratory of 2D Optoelectronics and Nanoelectronics (L2DON), Department of Materials Science and Engineering, Southern University of Science and Technology, 1088 Xueyuan Blvd, Shenzhen 518055, China; 12331184@mail.sustech.edu.cn (Y.L.); 12132085@mail.sustech.edu.cn (Y.W.); chaiyj@sustech.edu.cn (Y.C.)

**Keywords:** black phosphorus, MoS_2_, graphene, transistor, 2D electronics, reliability improvement

## Abstract

The last decade has seen dramatic progress in research on FETs with 2D channels. Starting from the single devices fabricated using exfoliated flakes in the early 2010s, by the early 2020s, 2D FETs being trialed for mass production and vertical stacking of 2D channels made by leading semiconductor companies. However, the industry is focused solely on transition metal dichalcogenide (TMD) channels coupled with conventional 3D oxide insulators such as Al_2_O_3_ and HfO_2_. This has resulted in numerous challenges, such as poor-quality interfaces and reliability limitations due to oxide traps. At the same time, the alternative routes for 2D FETs offered by laboratory (LAB) research have not been appreciated until now, even though the use of the native oxides of 2D channels has recently resulted in the first 2D FinFETs. Considering the research progress achieved in the last decade, from this perspective, we will discuss the main challenges for industry integration of 2D FETs and also suggest possible future steps which could propel these emerging technologies towards market applications.

## 1. Introduction

The scaling of Si technologies driven by Moore’s law has resulted in enormous progress towards making our electronics cheap and efficient in the last decades [1]. However, modern integrated circuits have recently approached their scaling limits. For instance, the typical channel lengths for the sub-2 nm nodes which are expected to come into play in the next few years will be below 12 nm [2], and further scaling would result in degraded performance due to short-channel effects. Although these limitations could be addressed by making Si channels as thin as a few nanometers, this appears to be impractical due to dramatically reduced carrier mobility [3]. Thus, naturally thin 2D semiconductors present the most feasible option for continuing the scaling of modern electronics and extending the life of Moore’s law [4,5]. Luckily, considerable progress in LAB research on FETs with 2D channels has been achieved exactly at the time when it is urgently needed, with recent demonstrations of large-area growth of TMD channels by chemical vapor deposition (CVD) [6,7] for trial circuits [8] and the discovery of alternative gate insulators such as CaF_2_ [9], STO [10] and native oxides of 2D channels [11,12].

**Figure 1:**
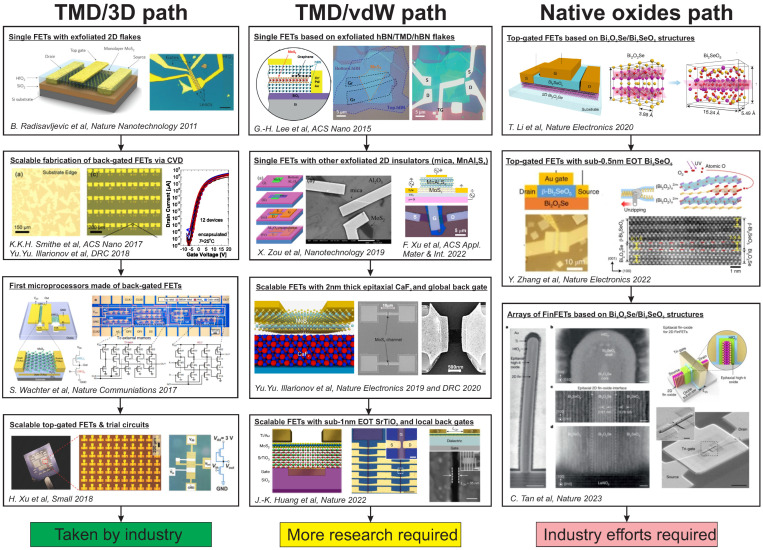
Key milestones of the three major paths of logic 2D FET technologies achieved in LAB research. While TMD/3D devices are already under trial industry processing, TMD/vdW FETs require more research, in particular in top gate integration. The devices with native oxides have recently reached the point of breakthrough towards FinFETs based on the Bi_2_O_2_Se/Bi_2_SeO_5_ system and thus urgently need industry attention to assess their future potential. Reproduced with permissions from [6,7,8,9,10,11,12,17,25,26,27].

Following the initial demonstration of the field effect in graphene [13], research attention has switched to real 2D semiconductors, with TMD channels being the most obvious choice for enabling logic FETs due to their sizable bandgaps [14] and the wide availability of controllable large-area CVD growth methods [15]. In contrast, zero-bandgap graphene is mostly interesting for other applications such as optoelectronics and sensors. MXenes lack large-area growth techniques and do not enable FET performance comparable to TMD devices, though they are still attractive as sensors [16]. In Figure 1, we summarize the three main paths and key milestones of the LAB research performed in the last decade. Starting from the first proof-of-concept studies of the early 2010s [17], the combination of an MoS_2_ [8,18,19] or another TMD [20,21] channel and a 3D oxide like SiO_2_ [22], Al_2_O_3_ [19] or HfO_2_ [23] has become common for almost any 2D FET fabricated in research labs. As a result, this TMD/3D path has progressed all the way from single prototype devices made of exfoliated flakes to trial circuit integration [7,8]. This has become possible due to the breakthrough progress achieved in the second half of the 2010s, first in the growth of large-area TMD films by CVD [6,15], and then also in the deposition of high-k oxides onto the TMD channels by atomic layer deposition (ALD) [7]. Remarkably, many LAB TMD/3D FETs exhibit very promising performance with near-ideal subthreshold swing [23] and on/off current ratios up to 10^10^ [24] and thus seem suitable for commercial applications at first glance [5].

Despite the enormous progress in TMD/3D FETs, already at the very beginning it was known that 2D insulators may be more suitable than widely available 3D oxides as they form van der Waals (vdW) interfaces with TMD channels [18,28]. This has shaped the second TMD/vdW path for 2D FET technologies. Here, major attention has been paid to hBN, which has finally appeared as not suitable for scaling due to mediocre dielectric properties [29] even though considerable progress in CVD growth of hBN [30,31] has been achieved as compared to the initially used hBN/TMD/hBN flakes [25]. Although the community has started to look into beyond-hBN 2D insulators, the first device demonstrations with mica [26] and MnAl_2_S_4_ [32] were still limited to exfoliated flakes. Thus, this path has reached the scalable production of MoS_2_ FETs with ionic CaF_2_ crystals which form quasi vdW interfaces with 2D channels [9,33,34]. While FETs with just 2 nm thick CaF_2_ films have demonstrated promising performance, these were only back-gated devices with a channel length down to 50 nm [34]. The same problem also applies to FETs with ultra-high-k (permittivity around 300) SrTiO_3_ (STO) insulators, even though, in that case, local back gates are created, and the developed transfer method for STO may hypothetically allow top gate integration [10]. As a result, more research on the scalable growth of beyond-hBN vdW insulators or deposition of fluorides on top of TMDs is still required to continue the TMD/vdW path and make a clear conclusion about its future potential.

Given the obvious problems of the TMD/3D and TMD/vdW paths, most recently, the research community has started to look back into the native oxide approach, which is the key foundation of Si technologies owing to its perfect Si/SiO_2_ interface. However, initially obtained native oxides of TMDs such as MoO_3_ [35] and WO_3_ [36] suffered from poor dielectric stability owing to their non-stoichiometric structure; thus, they were not suitable for device applications despite a number of available oxidation strategies for TMDs [37]. Luckily, a much better result was obtained using a more exotic 2D semiconductor, Bi_2_O_2_Se, which can be controllably oxidized into its native oxide Bi_2_SeO_5_ while keeping an atomically flat vdW interface and high crystallinity [11]. This approach has resulted in excellent top-gated FETs with low gate leakage currents even for sub-0.5 nm equivalent oxide thickness (EOT) [27]. Furthermore, most recently, even the first 2D FinFETs assembled into arrays [12] have been fabricated using the Bi_2_O_2_Se/Bi_2_SeO_5_ system obtained by oxidizing wafer-scale arrays of epitaxially grown vertical Bi_2_O_2_Se fins. This makes the native oxide path of 2D FETs very attractive for the industry.

**Figure 2:**
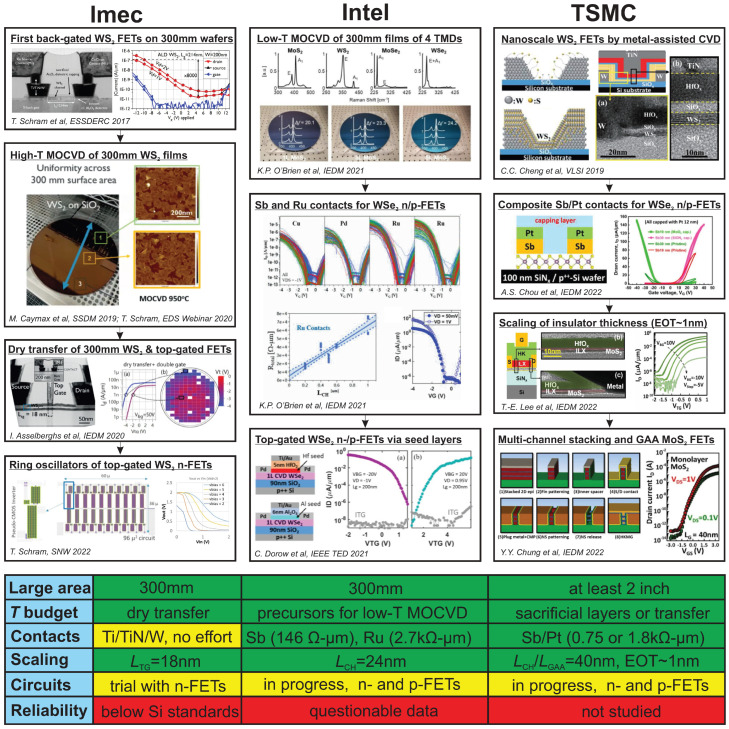
Key milestones of the recent efforts at integrating TMD/3D FETs achieved by imec, Intel and TSMC in the last few years. The table in the bottom shows that all three companies have addressed the initial issues and approached the stage of trial circuit integration, even though imec has not demonstrated mature p-FETs so far. However, reliability of available prototypes of FAB TMD/3D FETs is either far below Si standards or not well understood. Reproduced with permissions from [38,41,42,43,44,46,48,49,50].

Appreciating the above advances made by our research society, the industry started to look into the FAB integration of 2D FETs back in 2017, when imec reported the first WS_2_ films grown by CVD and ALD on 300 mm wafers [38]. Up to now, imec, Intel and TSMC have shaped their own paths for 2D FETs, as shown in Figure 2. Remarkably, they all rely solely on the TMD/3D strategy since the widely available high-k oxides are more suitable for the process lines developed for Si technologies. These leading companies have already addressed the most obvious initial issues of LAB-to-FAB transition, which are the controllable growth of large-area 2D films and the need to match the CMOS thermal budget of 450 °C [39]. For instance, imec uses their mature dry transfer method for large TMD films grown by metal-organic CVD (MOCVD) on sacrificial substrates at up to 1000 °C [40,41]; Intel employs MOCVD on target substrates, with high quality of at least four TMDs achieved by proper selection of metal-organic precursors even at 300–400 °C [42]; and TSMC has several options including metal-assisted CVD [43] and transfer methods [44]. Also, the three leaders have demonstrated scalable fabrication of top-gated TMD/3D FETs down to nanoscale dimensions via ALD growth of high-k oxides on TMD channels [41,43,45], and TSMC has recently made a breakthrough towards a gate all around (GAA) MoS_2_ FETs for multi-channel stacking [44]. In recent studies by Intel [42] and TSMC [46], the contact resistance was reduced below 1 kΩ by using Sb, Ru or composite Sb/Pt contacts. However, a more extensive look into the techniques for making clean contacts known from LAB research [47] may be still required. While contact metals and seed layers used for ALD growth of high-k oxides offer excellent opportunities for obtaining n- and p-FETs using the same TMD channel [46,48], Intel and TSMC have approached trial complementary integration of their devices. Furthermore, imec, which has not yet reported top-gated p-FETs, still demonstrated ring oscillators made of WS_2_ n-FETs [49], which may open a way to pseudo CMOS integration of 2D FETs in future. It is also important that all the manufacturers have demonstrated FETs with channel lengths scaled down to tens of nanometers, and TSMC has invested specific efforts into the scaling of the top gate insulator down to an EOT of about 1 nm [50]. However, it is still important to reproduce excellent performance parameters such as the subthreshold swing (SS) and mobility achieved in LAB devices (summarized in [29,51]) when moving to FAB devices, including new designs like GAA FETs. While this is expected to be possible via further optimization of FAB processes, the major fundamental issue which is still not addressed by the industry is the reliability of TMD/3D FETs. For instance, the first imec devices are far below Si standards [52], Intel has provided only fragmentary data [45] and no studies are available for TSMC devices.

**Figure 3:**
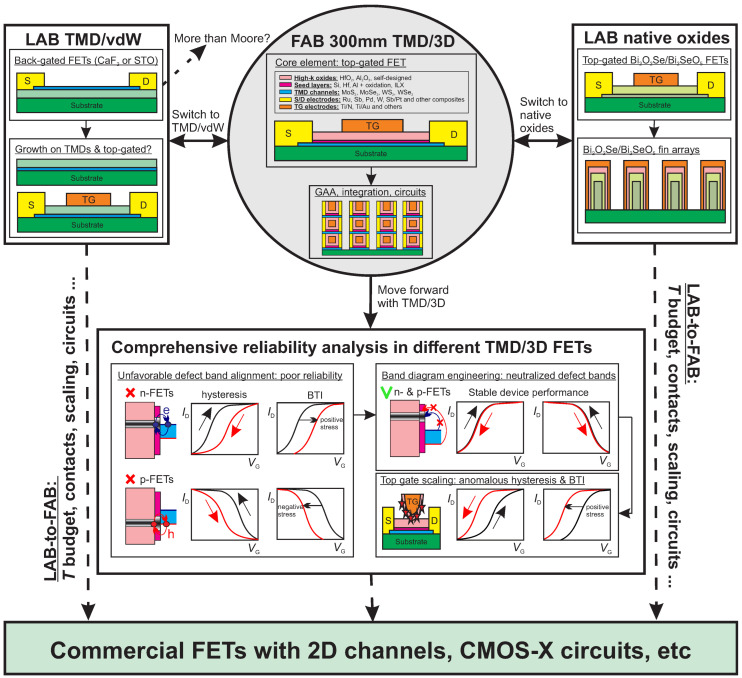
Schematic representation of possible future development of 2D FET technologies. TMD/3D path has already entered FAB lines and gained an extensive choice of materials for channels, insulators and contacts. However, comprehensive reliability analysis is still required, which should include analysis of the optimum choice of channel/oxide combinations to suppress the impact of oxide defect bands and also address the issues related to process-induced defects in FETs with nanoscale top gates. At the same time, native oxide and TMD/vdW FETs still require more research attention and a long LAB-to-FAB transition even though they may have some advantage over the TMD/3D devices in terms of their reliability.

Based on the present stage of research on 2D FETs and already achieved progress in their LAB-to-FAB transition, in Figure 3, we schematically illustrate possible future trends in the development of these new technologies. Considering the fast initial success of the TMD/3D strategy was based on decades-long experience with Si technologies, we are confident that this path selected by the industry will continue playing the key role in the foreseeable future. Thus, in the coming years, the semiconductor industry will continue using a top-gated TMD/3D FET as the core element in their studies. They will further extend the range of TMD channels suitable for large-area growth at reasonable temperatures and develop their custom high-k oxides and seed layers for ALD growth while keeping their composition a secret, e.g., TSMC’s ILX [50]. At the same time, apart from achieving high quality for the dielectric stacks, the goal will consist of scaling its EOT below 1 nm. Also, more options to vary the channel conductivity type by changing the chemical content of contacts and seed layers will be suggested by extending the range of available materials. This will pave the way towards more attempts at circuit integration of FAB 2D FETs, possibly including those made using vertical stacking and GAA device layouts [44].

However, with all the obvious advantages of the TMD/3D strategy selected by the industry, high-k oxides, known from Si technologies, are not naturally made for 2D materials. Although the industry has already mastered their ALD growth on top of various TMD channels with a quality good enough to separate the top gate from the channel, these oxides are still amorphous when grown in thin layers. As a result, both the high-k oxides and nm thick oxidized seed layers used for their nucleation on inert 2D surfaces should contain a sizable number of border traps [53]. By exchanging charges with the channel, these defects can cause hysteresis and bias temperature instabilities (BTI) of the gate transfer characteristics, thereby severely degrading the device reliability [51,54]. Thus, given our extensive experience with various LAB 2D FETs [9,24,54] and the first imec FAB prototypes of MoS_2_ FETs [52], we are confident that the progress of TMD/3D devices to mass production will include comprehensive reliability analysis. Considering that charge trapping is thermally activated [54], which results in a dramatic increase in BTI drifts at high temperatures, reliability tests will have to be performed at different temperatures in order to determine the safe operation conditions of 2D FETs. As schematically shown in Figure 3, the most feasible way to enhance the reliability of TMD/3D FETs should be based on band diagram engineering [51,55]. Therefore, we expect that the industry will finally use only those TMD/3D oxide combinations in which fundamental oxide defect bands [56] are energetically far from the conduction and valence bands of the channels, thereby increasing the energy barriers for charge trapping in both n- and p-FETs. Interestingly, long ago, a similar approach was tested by imec when using SiGe to suppress charge trapping in Si FETs [57]. Considering our recent results on the nanoscale imec MoS_2_ FETs showing counterclockwise hysteresis and abnormal BTI dynamics due to process-induced defects in scaled top gate stacks [52], we expect that, at the next stage, considerable attention will be paid to the further improvement of the processing techniques.

Still, despite our moderately optimistic view on the future of TMD/3D FETs, we expect that the most recent breakthrough towards Bi_2_O_2_Se/Bi_2_SeO_5_ FinFETs [12] will encourage the industry to make considerable investments in the native oxide strategy, including oxidation of TMDs [35,36]. This is not to exclude the possibility that, given the relative simplicity of the oxidation process and some similarity to the Si/SiO_2_ technology, in the coming years, this approach will be reproduced using FAB process lines. As for the TMD/vdW path, from today’s point of view, it should be highly demanded for More than Moore applications such as sensors [58] or photodetectors [59], which do not necessarily require top gates. However, given the recent progress in the epitaxial growth of crystalline CaF_2_ on silicene [60], we would not exclude the possibility that, sooner or later, competitive top-gated FETs will be also created using TMDs or even other 2D channels. It is also worth noting that the extensive findings on low-resistance contacts and vertical stacking gained for the TMD/3D FETs could be transferred to the two alternative paths if, in future, they reach the FAB integration stage. As for the reliability issues, owing to the crystalline nature of insulators, this problem is expected to be less sound for future TMD/vdW and native oxide FETs.

We also note that most of the challenges with the FAB integration of TMD FETs discussed above are also relevant to graphene devices. Although graphene cannot be used for logic FETs due to its zero bandgap, this material is attractive for optoelectronics, sensors and radio-frequency applications, which can be integrated in the same circuits and thus should be technologically compatible with FETs. Furthermore, graphene devices also contain channel/insulator interfaces and thus suffer from the same reliability issues which can be hopefully addressed by using crystalline insulators like CaF_2_ [61], or band diagram engineering if conventional 3D oxides are used [55]. Thus, graphene/3D and graphene/vdW paths could be developed similarly to TMD/3D and TMD/vdW paths, considering specific features of the large-area growth of graphene and relaxed requirements for insulator scaling for non-logic devices. Furthermore, the use of MXenes [16] in electronic devices would also require addressing most of the challenges discussed above if these materials approach FAB integration at a certain point.

In summary, we suppose that, sooner or later, the industry will find a way to address the existing reliability challenges of TMD/3D FETs and select suitable material combinations for mass production. This, however, does not exclude the possible parallel development of the two other strategies employing the native oxides of 2D channels and vdW insulators, which could benefit from the recent achievements made for TMD/3D devices. Furthermore, an avalanche breakthrough in the LAB-to-FAB integration of these alternative 2D FET technologies may be possible if leading semiconductor companies start investing in them as much as they have been doing for devices with conventional 3D oxides in the last few years.

## Data Availability

Data are contained within the article.
